# Unveiling vocal profiles in adolescent anorexia nervosa: a Software Based, Multiparametric Analysis

**DOI:** 10.1007/s00787-024-02524-5

**Published:** 2024-07-22

**Authors:** Jacopo Pruccoli, Giulio Rocco di Torrepadula, Luca Bergonzini, Valentina Genovese, Antonia Parmeggiani

**Affiliations:** 1 IRCCS Istituto delle Scienze Neurologiche di Bologna, UOC Neuropsichiatria dell’Età Pediatrica, Centro Regionale per i Disturbi della Nutrizione e dell’Alimentazione in età evolutiva, Bologna, Italy; 2 Dipartimento di Scienze Mediche e Chirurgiche (DIMEC), Università di Bologna, Bologna, 40126 Italy; 3 Corso di Laurea di Logopedia, Università di Bologna, Campus Ravenna, Bologna, Italy; 4 Direzione Infermieristica e Tecnica, Ausl Romagna, Faenza, Italy

**Keywords:** Voice, Dysphonia, Anorexia nervosa, Feeding and eating disorders, Adolescents, Laryngopharyngeal reflux.

## Abstract

Dysphonia, characterized by disturbances in voice quality and modulation, has been sporadically observed in individuals with Anorexia Nervosa (AN), potentially stemming from both organic and psychopathological factors. This study seeks to employ software-based voice analysis to compare the voices of girls with AN to those of female healthy controls (HC). Case-control study adopting “Praat” software to assess voices. Various parameters, including Acoustic Voice Quality Index (AVQI), Fundamental Frequency (F0), Yanagihara’s Spectrographic Dysphonia Classifications, and “GIRBAS” perceptual qualitative voice rating, were investigated. Participants completed questionnaires for Vocal Fatigue Index (VFI) and the Reflux Symptoms Index (RSI). Puberty-related voice spectrum changes were considered, and Bonferroni-corrected BMI-adjusted Analyses of Covariance (ANCOVAs) were conducted. The study enrolled 15 girls with AN and 23 girls with HC. AN patients demonstrated greater impairment in voice tiredness/voice avoidance (VFI-1, *p* < 0.001), vocal physical discomfort (VIF-2, *p* = 0.002), and rest as alleviation (VFI-3, *p* = 0.012). Reflux-related scores were higher in AN (*p* < 0.001). Differences were observed in voice quality (AVQI) (*p* = 0.001), and GIRBAS scales showed alterations in multiple parameters. Spectrograms documented more frequent pathological findings in AN patients (*p* = 0.021). No difference was observed in Fundamental Frequency. These group (AN/HC) differences were independent of weight measures. This study is the first to connect voice irregularities in AN by employing standardized, non-invasive tools and accounting for weight-related factors. Young AN patients demonstrated substantial voice quality changes and heightened self-reported symptoms. Future research should expand on these findings with prospective designs and invasive investigations.

## Introduction

Anorexia Nervosa (AN) is a Feeding and Eating Disorder (FED) characterized by restriction of energy intake up to self-starvation, intense fear of gaining weight, and distorted body image perception, resulting in emotional, behavioral, and social impairment [1]. The incidence of AN peaks during adolescence in both men and women and AN carries the highest mortality rate of all mental health conditions [2] due to cardiovascular, metabolic, or gastrointestinal involvement in the disease [3]. Psychiatric and somatic comorbidities also determine a relevant impairment in terms of health-related quality of life (HRQoL) among patients with AN [4]. Vocal involvement is not routinely accounted for among the complications of AN since few authors have described the impact of AN and other FED on the vocal apparatus of patients [5, 6].

Voice disorders encompass a broad spectrum of conditions that alter the vocal quality, pitch, loudness, or vocal effort causing dysphonia [7]. To date, no single framework provides clear-cut labels and definitions for all voice disorders across the spectrum. A broad division into organic, functional, and muscle tension voice disorders is currently accepted for classification [8]. Voice disorders affect all ages, particularly the pediatric population and the elderly: estimates indicate 23.4% of children develop dysphonia, with higher prevalence among those in the 8- to 14-years age range, primarly due to vocal overuse and phonotrauma [9]. The impact of dysphonia on HRQoL is substantial across all ages, since affected patients may experience limitations in their activities, lifestyle changes, and, ultimately, social isolation depending on the severity of symptoms [8, 10].

The relationship between FED and voice disorders has been addressed over the past years, mainly concerning Bulimia Nervosa (BN). Several authors hypothesized patients with BN may experience dysphonia because of purging behaviors. Indeed, these may cause the detrimental exposition of vocal folds to gastric contents due to vomiting or lower esophageal sphincter weakness with laryngopharyngeal reflux [11]. Scant evidence is available concerning the relationship between AN and voice disorders: Lawrence and colleagues observed a higher prevalence of voice disorders among adult patients with AN (23%) than with BN (18%) [6]. Garcia-Santana and collegues found that adolescent girls with AN have a higher-pitched voice than healthy controls [12]. Maciejewska and colleagues found adolescent girls with AN had inadequate growth of laryngeal muscles and lower fundamental frequency (F0) than healthy controls. The authors speculate endocrine dysfunction, along with prolonged starvation, may lead to these structural and functional changes in the vocal apparatus of adolescents with AN [5].

So far, little is known about the relationship between voice disorders and AN, and what is known mostly overlooks issues of the AN purging subtype. The primary aim of the present study was to describe the occurrence of dysphonia in adolescent girls with AN, compared to age- and sex-matched healthy controls (HC). A further objective was to characterize the degree of vocal alteration through a standardized non-invasive qualitative and quantitative analysis by studying changes in vocal pitch, timbre, intensity, duration, or proprioceptive sensations.

## Methods

### Study design and participants

This prospective, case-controlled study occurred in a third-level Regional Center for FED in Children and Adolescents. The study was approved by the local ethical committee (code NPI-ANVOX2022) and it was not sponsored or funded by any company. Informed consent was obtained from all patients, HC, and their caregivers, following the guidelines outlined in the Declaration of Helsinki and the approved study protocol. No additional filters were applied during the selection process.

The idea for this study stems from recognizing the significance of a multidisciplinary approach to FED. Speech-language pathologists (SLPs) bring expertise in communication, treating voice disorders, and enhancing vocal strength and health, crucial for daily coping. Within our Child Neuropsychiatry department, SLPs are already integrated, playing a pivotal role in teams addressing developmental issues like dysphagia, language, and cognitive disorders in children. The interdisciplinary collaboration between child neuropsychiatrists and SLPs in the department likely prompted the investigation into voice-related parameters, despite it not being the initially targeted population. This dynamic interaction underscores the importance of continuous feedback loops in clinical settings, potentially leading to insights with broader implications for addressing voice-related issues in similar populations.

The research was conducted between February and May 2023, by considering patients hospitalized for AN at the study center (cases), as well as HC. Hospitalization was defined as either inpatient or day hospital (DH) treatment. The DH treatment program for FED patients is structured similarly and equally intensive as inpatient treatment. The hospital program in our center involves a comprehensive multidisciplinary approach that includes psychological, medical, and nutritional interventions. All included patients were subjected to the same multidisciplinary program that was conducted by the same team, in the same Center, and adhered to international clinical guidelines [13, 14]. HC were recruited from a local basketball team belonging to an amateur sporting association.

Inclusion criteria for cases were (1) a current diagnosis of AN according to specific DSM-5 criteria [1]; individuals with Atypical AN were not included; both subtypes of AN (restrictive, with binge/purging, or both) were admitted; (2) an age range of 11–17 years of age; (3) female sex. Notably, criterion A for a DSM-5-based diagnosis of AN requires “Significantly low weight”, defined as “a weight that is less than minimally normal or, for children and adolescents, less than that minimally expected” [1]. Thus, all the included cases (AN) were underweighted.

Inclusion criteria for HC were (1) no diagnosis of AN, FED, or other psychiatric disorders according to DSM-5; (2) age range of 11–17 years of age; (3) female sex; (4) body weight > 10° centile for age and sex. The 10° centile cutoff was adopted, according to the published criteria of the European Society for Child and Adolescent Psychiatry [15].

All patients were following the same multidisciplinary program for AN according to clinical international guidelines.

The study was conducted entirely in Italian. All questionnaires and interviews were administered in Italian. Both the participants with AN and the HC were of Italian origin, and fluent into the Italian language. All measurements were adapted to Italian standardizations, except for the VFI, which adhered to American standards.

### Assessment methods

All measurements and clinical interviews were administered to both the AN and the HC groups.

#### Clinical evaluation

A clinical interview with child neuropsychiatrists experienced in the field of AN was carried out and information about AN diagnosis (“typical” AN, restrictive or binge/purging subtypes), BMI, comorbidities, and current treatment was reviewed. Participants’ BMI was compared to a sex and age-standardized estimate of mean BMI to determine its variance from the general population (%BMI). The use of %BMI represents a standard in the clinical practice with children and adolescents with AN, since BMI may represent a less reliable clinical indicator in these age groups [16].

#### Diagnosis of AN and psychiatric comorbidities

The inclusion criteria required the patients to have a diagnosis of AN, and no diagnosis of AN (or any other FED) for HC. To ascertain this, all the included cases and HC underwent a standardized assessment, based on the Eating Disorder Examination (EDE) [17].

The potential presence of mental health diagnoses/comorbidities was investigated with a clinical interview, based on the Schedule for Affective Disorders and Schizophrenia for School-age Children and Lifetime Version (K-SADS-PL), in its Italian version updated for use with the DSM-5 [18]. Both assessments were performed by a child and adolescent neuropsychiatrist expert in the field of FED.

#### GER symptoms

Participants were asked to fill out the Reflux Symptom Index (RSI) questionnaire to investigate the potential occurrence of LPRD (extraesophageal variant of GERD) symptoms, given as a paper-pen form. The questionnaire explores symptoms of reflux through 9 items, each of which can be scored from zero (no complaints) to five (severe complaints), with of 13 or higher suggestive of LPR [19].

#### Evaluator’s based and subjective assessment of voice quality

Vocal assessment was then led by a trained SLP. Evaluator-based (1, 2) and subjective (3) voice measures were collected.

1. The perceptual evaluation of dysphonia was led through the Grade, Instability, Roughness, Breathiness, Asthenia, and Strain (GIRBAS) scale. This scale allows examiners to perform a qualitative assessment by scoring each parameter from 0 (Euphonia) to 3 (severe vocal impairment) [20].
2. The aerodynamic analysis of phonation is based on the close anatomical and functional connection between the larynx and the bronchopulmonary system. The four fundamental aerodynamic features are the airflow velocity at the level of the glottis, the subglottic pressure, the supraglottic pressure, and the glottic impedance. The simplest aerodynamic parameter of voice is the maximum phonation time (MPT) expressed in seconds. MPT is measured by having the patient sustain the vowel /a/ for as long as possible after a deep inspiration, at a spontaneous and comfortable pitch and intensity [21]. It is measured in seconds (s), and in this study was gathered using a hand chronometer.
3. The Vocal Fatigue Index (VFI) is a self-report tool to assess the symptom profile of vocal fatigue (VF) and its associated behavioral aspects. Three elements are considered: (1) Tiredness and Avoidance; (2) Physical Discomfort; (3) Symptom Improvement (or Lack of) with Rest. Respectively, scores ≥ 24, ≥7, ≤ 7 indicate vocal fatigue [22]. In this study, the authors put out a comparison between AN and HC, because no Italian standardization is still available; although they chose to reference the American standardization which states that a pathological score can be referenced to a score ≥ 24 for Sect. 1, ≥ 7 for Sect. 2, ≤ 7 for Sect. 3.

#### Software-based assessment of voice quality

The objective analyses of voice quality were based on “*Praat*” software [23], commonly to analyze, synthesize, and manipulate vocal tracks. It was created by Paul Boersma and David Weenink from the Institute of Phonetic Sciences at the University of Amsterdam. In this study, the vocal analysis component was primarily utilized, enabling the identification of the fundamental frequency and vocal quality through the spectrogram and Acoustic Voice Quality Index. During the assessment, both the clinician and the patient were situated in a quiet environment with minimal background noise. A Samson Meteor Mic microphone was positioned approximately 30 centimeters from the patient’s mouth, and no disruptions were encountered during the recording process. Three main Praat-related parameters were considered for this study:

1. The Fundamental Frequency (F0) indicates the number of glottal openings and closing per second and represents the pitch of the glottic signal. From a physiological standpoint, the value of the signal’s frequency depends on numerous input factors (subglottic, glottal, and supraglottic), which are, in turn, influenced by constitutional, age, and gender-related factors, among others. However, variations in F0 typically revolve around an average value known as the modal fundamental frequency. Its value ranges from 225 to 440 Hz in children, from 175 to 245 Hz in adult females, and from 105 to 160 Hz in adult males. The decision to utilize an average F0 is rooted in the premise that individuals with AN often exhibit a larynx morphology and positioning akin to that of children. F0 is affected not only by vocal cord dimensions but also by larynx position, with the primary objective being to discern general indicators of laryngeal status rather than specific tissue conditions of the vocal folds.
2. The Acoustic Voice Quality Index (AVQI) is an acoustic measure of vocal quality used in clinical and research settings. It was developed building upon existing models, but it stands out as the only one capable of providing the severity grade of dysphonia for both sustained vowels (SV) and continuous speech (CS). To achieve this, AVQI combines six acoustic parameters from the two sections, SV and CS, which are related to pitch and frequency variations in the sample. This index is positively correlated with the perceptual judgment of vocal quality. The cutoff score is not unique and can be considered either 2.33 or 3, depending on the desired level of sensitivity and specificity [24].
3. The Spectrogram is a commonly used graph to represent patients’ voices. It displays frequency on the y-axis, time on the x-axis, and amplitude by the darkness of lines. Two types of spectrograms exist: wideband and narrowband. Wideband filters (e.g., 300, 500 Hz) offer excellent time resolution for analyzing formants. Narrowband filters (e.g., 45, 50 Hz) allow precise analysis of individual harmonics. A qualitative description of the graph includes observations of noise, air leakage, vocal breaks, and harmonic amplitudes. Alternatively, Yanagihara’s classification has been adopted. This system employs the emission of the single vowel /a/ for spectrum analysis instead of multiple vowels, providing severity classes (0 to 3) and two additional qualitative parameters: diplophonia (d) and vocal tremor (t) [25]. For this study, the description of the spectrogram was standardized using Yanagihara’s scale, following Ricci-Maccarini’s revision [26], which states that Class 0 describes a spectrum without any signs of noise or other alteration, Class 1 relates to a spectrum with noise over 2000 Hz with the possibility of recognizing the harmonics, Class 2 refers to harmonics that can be distinguished until 2000 Hz, Class 3 describes a spectrum with no harmonics over 500 Hz.

### Statistical analysis

Descriptive analyses were provided for the entire sample, as well as for patients and HC separately. Shapiro–Wilk’s and Levene’s tests were used to assess the normality of data distribution and homogeneity of variance. Possible differences between the two groups were considered and were investigated with t-tests for continuous variables (Mann–Whitney when required) and chi-square tests for nominal variables.

Differences in the demographic and clinical variables (before vocal assessments) between the two groups were considered as potential confounders. Vocal measurement scores were compared between the two groups, and all the analyses were controlled for potential confounders. To do so, multiple covariance (ANCOVA) analyses were conducted, using each vocal measurement score as a dependent variable, group status (AN/HC) as a categorical independent variable, and potential confounders as independent variables. To correct the resulting ANCOVAs for the potential effect of running multiple comparisons between the two groups, a Bonferroni correction was applied to all the analyses.

Pearson’s correlations, linear, and multiple regression were run to explore significant relationships between variables. Correlations were both run within the whole sample and within patients with AN only. The significance level was set at 0.05, and all tests were two-tailed. All the statistical analyses were conducted with SPSS, version 26.0 for Windows.

## Results

### Demographic and clinical features

Over the study period, 15 adolescent girls with AN and 23 HC were enrolled. All the included individuals with AN presented a restrictive subtype.

All individuals, as an inclusion criterion, with AN and HC were females and the mean age at the assessment was comparable between the two groups: 14.9 ± 1.2 and 14.7 ± 1.4 years of age, respectively (*p* = 0.511; d=-0.205). Conversely, as an inclusion criterion, individuals with AN showed significantly lower BMI (15.4 ± 1.2 vs. 19.8 ± 2.1 kg/m^2^, *p* < 0.001; d = 2.461) and %BMI than HC (76.8% ± 6.5 vs. 99.9% ± 10.1, *p* < 0.001; d = 2.592). %BMI ranged between 64.1% and 84.8% for the AN group and between 85.1% and 128.6% for the HC group. Given the statistically significant difference in weight measures between the two groups, weight measures (considered as %BMI, as documented in the Methods section) were retained as a potential confounder and included in the following comparisons between the two groups.

No one among individuals with AN or HC was undergoing any gastroprotective medication (e.g., proton pump inhibitors) at the moment of the assessment. All individuals denied habitual alcohol and tobacco use or the presence of GERD symptoms, as they are often related to gastroesophageal and laryngeal diseases. However, 13 individuals with AN were receiving selective serotonin reuptake inhibitors (SSRIs) and 3 of them were prescribed olanzapine. All therapies had been consistently administered for at least 3 months prior to the assessment.

Two individuals were singers. They were both located in the group with AN; one girl was an amateur singer, with no history of lessons, and the other one used to take classes before hospitalization (about four months before enrollment).

### Reflux symptoms

As stated, RSI is a self-reported questionnaire used to enlighten largo-pharyngeal reflux symptoms; a score over 13 is a pathological score. The mean RSI scores were 15.1 (± 7.1) for the group with AN, and 1.6 (± 1.6) for HC. Individuals with AN showed significantly higher scores at the RSI than HC (*p* < 0.001; d=-2.928). Specifically, RSI among individuals with AN ranged from 4 to 27, and 11 patients (73.3%) had an RSI of 10 points or more; conversely, 100% of HC had a subthreshold RSI, ranging from 0 to 5 points (X^2^ = 23.74, *p* < 0.001). Pearson’s correlation showed a significant correlation between %BMI and RSI scores, (*r*=-0.661, *p* < 0.001; z=-0.795) %BMI accounting for 44% of the variance in RSI scores. Linear regression showed that %BMI can significantly predict RSI scores (F(1, 36) = 27.972, *p* < 0.001) using the following regression equation: RSI score = 40.669+(-0.371*%BMI). There was a significant relationship between AN diagnosis and RSI scores after controlling for %BMI according to ANCOVA, with a large effect size (F (1, 35) = 27.308, *p* < 0.001; η2 = 0.247). No significant correlation was documented when assessing potential correlations between RSI scores and %BMI in patients with AN only (*r*=-0.042, *p* = 0.882; z = 0.289).

### Vocal assessment and voice measures

As reported, GIRBAS is a voice perceptual scale, based on different types of alterations: Grade, Instability, Roughness, Breathiness, Asthenia, and Strain. The authors highlighted the differences between euphonic voice (0) and dysphonia (≥ 1). Results from the perceptual evaluation of dysphonia using the GIRBAS scale are summarized in Table 1. Neither individuals with AN nor HC showed moderate or high degrees of vocal impairment in any of the items; therefore, the frequency of mild degree impairment between the two groups was considered. After a Bonferroni correction, individuals with AN showed a statistically higher frequency of mild impairment in the grade of global dysphonia, vocal instability, and breathiness.

**Table 1:** Frequency of mild scores on the GIRBAS Scale among the two groups, compared with Chi-squared tests

	AN (*n* = 15)	HC (*n* = 23)	Statistics
Grade	15 (100%)	13 (56.5%)	**X**^**2**^ **= 8.851**,*p* = 0.003*,** phi = 0.483**
Instability	13 (86.7%)	6 (26.1%)	**X**^**2**^ **= 13.328**,*p* < 0.001*,** phi = 0.592**
Roughness	7 (46.7%)	4 (17.4%)	X^2^ = 3.783, *p* = 0.052, phi = 0.316
Breathiness	12 (80%)	8 (34.8%)	**X**^**2**^ **= 7.446**,*p* = 0.006*,** phi = 0.316**
Asthenia	3 (20%)	1 (4.3%)	X^2^ = 2.362, *p* = 0.124, phi = 0.249
Strain	0 (0%)	0 (0%)	*p* = 1.000

*Note* Bonferroni-corrected level of significance: *p* = 0.050/6 = 0.0083. Abbreviations: AN: Anorexia Nervosa; HC: Healthy Controls. Statistically significant differences are marked in bold and with an asterisk (*)

Tables 2 and 3 describe comparisons of the voice measures obtained through the vocal assessment of individuals with AN and HC.

The mean MPT, measured in seconds by holding a sustained /a/, in the two groups was found to be comparable, with a slight difference in the mean duration at the edge of statistical significance (*p* = 0.055, Table 2). However, pathologic MPT (MPT < 10 s) was exclusively found among individuals with AN (*p* = 0.025, Table 3). No significant correlation between MPT and %BMI was found. MPT was still comparable between the two groups after controlling for %BMI (F(1,35) = 3.926, *p* = 0.055).

The mean Vocal Fatigue Index (VFI) scores were higher among patients with AN than among HC concerning all three questionnaire subscales (*p* < 0.001, *p* = 0.002, *p* = 0.012, Table 2). Pathologic scores were more frequent among individuals with AN than HC for both VFI-1 (Tiredness and Avoidance) and VFI-2 (Physical Discomfort) (*p* < 0.001, *p* = 0.003, Table 3), but the frequency was comparable – on the edge of statistical significance – between the two groups for VFI-3 (Symptom Improvement with Rest) (*p* = 0.055). According to ANCOVA, a significant relationship between AN diagnosis and VFI scores was found after controlling for %BMI (VFI-1: F(1,35) = 16.566, *p* < 0.001, η2 = 0.206; VFI-2: F(1,35) = 7.111, *p* = 0.012, η2 = 0.124; VFI-3: F(1,35) = 3.884, *p* = 0.057, η2 = 0.091).

Fundamental frequency (F0), ranges typically from 225 to 440 Hz in children, from 175 to 245 Hz in adult females, and from 105 to 160 Hz in adult males, was comparable between the two groups in our study, when assessed by analyzing vocal records using the Praat software (*p* = 0.062, Table 3). No statistically significant difference between individuals with AN and HC on F0 resulted in a one-way ANCOVA after controlling for %BMI (F(1,35) = 0.341, *p* = 0.563; η2 = 0.010).

The software analysis showed a significantly different Acoustic Voice Quality Index (AVQI), an overall index of voice quality, and mean scores between the two groups, with individuals with AN having higher AVQI than HC (*p* = 0.001, Table 2). Anyway, the frequency of over-threshold AVQI alterations (AVQI > 2.33) did not statistically differ between individuals with AN and HC (*p* = 0.087, Table 3). A significant correlation was found between AVQI and %BMI (*r*=-0.352, *p* = 0.030, z=-0.368). Multiple linear regression using backward data entry did not show that %BMI can predict AVQI score. At a one-way ANCOVA, a significant relationship between AN diagnosis and AVQI scores was found after controlling for %BMI (F(1, 35) = 7.019, *p* = 0.012, η2 = 0.146). When assessing potential correlations between AVQI and %BMI in patients with AN only, no significant correlation was documented (*r*=-0.029, *p* = 0.918; z = 0.288).

**Fig. 1:**
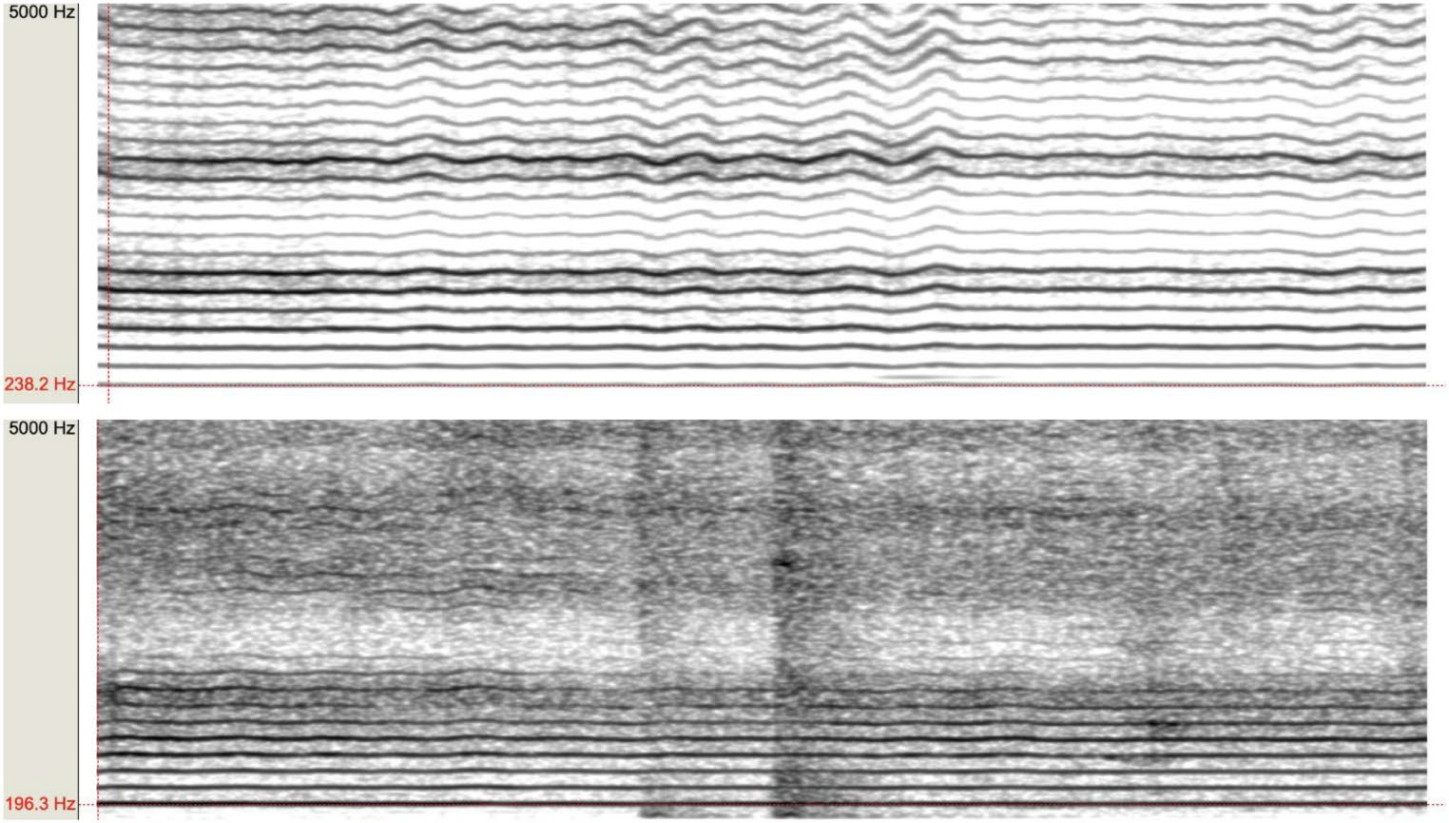
Compared spectrograms for an included healthy control (upper) and an included individual with Anorexia Nervosa (bottom). Notes: The spectral description using Yanagihara’s classification identifies three distinct classes, ordered based on increasing severity. Class 0 refers to a spectrum within the normal range (absence of noise or the presence of components that do not predominate over harmonic components). Class 1 indicates a mild alteration (presence of noise components, which predominate over harmonic components beyond 2000 Hz; however, harmonics are still present even beyond 2000 Hz). Class 2 is associated with a moderate alteration (presence of noise in the spectrum; harmonics are present only up to 2000 Hz), and Class 3 was not assigned. The spectrogram displays the formants that make up the voice based on the frequency range (x-axis) and time (y-axis), outlining their intensity through shading. The first formant is the fundamental/modal frequency F0, and all subsequent formants are multiples of the first one. The upper graph, obtained from an included healthy control, displays harmonics throughout the spectrum, with no evidence of noise (shown as mist). The graph on the bottom, related to an included patient with Anorexia Nervosa, reveals noise beyond 2000 Hz, a frequency beyond which harmonics are no longer distinguishable, and only noise is observed

Spectral analysis of vocal records obtained from individuals with AN and HC showed both groups similarly fit Yanagihara’s classes after Bonferroni correction (Tables 2 and 3). Differences in the spectrogram for two included individuals (one HC and one with AN) are reported in Fig. 1.

**Table 2:** Voice measures of the included sample, compared with independent samples T-tests, and Chi-squared tests for Yanagihara’s classes

Variables	AN (*n* = 15)	HC (*n* = 23)	Statistics
F0 (Hz)	214.7 ± 17.6	210.5 ± 29.0	t= -0.502, *p* = 0.062, d=-0.167
AVQI	3.4 ± 1.0	2.3 ± 0.9	**t= -3.578**,*p* = 0.001*,** d=-1.187**
MPT (sec)	15.3 ± 6.0	17.4 ± 2.6	U = 236.500, *p* = 0.055, d = 0.496
VFI-1	20.7 ± 10.5	4.3 ± 4.0	**U = 27.500**,*p* < 0.001*,** d=-2.273**
VFI-2	4.3 ± 3.3	0.8 ± 1.1	**U = 71.500**,*p* = 0.002*,** d=-1.583**
VFI-3	5.9 ± 3.3	2.9 ± 3.3	**U = 89.500**,*p* = 0.012*,** d=-0.924**
Yanagihara Class 0 Class 1 Class 2	2 (13.3%) 7 (46%) 6 (40%)	9 (39.1%) 12 (52.2%) 2 (8.7%)	X^2^ = 2.937, *p* = 0.086 X^2^ = 0.110, *p* = 0.739 X^2^ = 5.353, p = 0.021

*Note* Bonferroni-corrected level of significance: *p* = 0.050/3 = 0.016. Abbreviations: AN: Anorexia Nervosa; HC: healthy controls; VFI = Vocal Fatigue Index; AVQI = Acoustic Vocal Quality Index; MPT = maximum phonation time; F0 = fundamental frequency. Statistically significant differences are marked in bold and with an asterisk (*)

**Table 3:** Frequency of impaired voice measures at the vocal assessment, compared with Chi-squared tests

Variables	AN (*n* = 15)	HC (*n* = 23)	Statistics
AVQI > 2.35	13 (86.7%)	14 (60.9%)	X^2^ = 2.938, *p* = 0.087, phi = 0.536
MPT < 10 s	3 (20%)	0 (0%)	X^2^ = 4.994, *p* = 0.025, phi = 0.363
VFI-1 > 24	6 (40%)	0 (0%)	**X**^**2**^ **= 10.925**,*p* < 0.001*,** phi = 0.278**
VFI-2 > 7	5 (33.3%)	0 (0%)	**X**^**2**^ **= 8.828**,*p* = 0.003*,** phi = 0.482**
VFI-3 < 7	10 (66.6%)	21 (91.3%)	X^2^ = 3.667, *p* = 0.055, phi=-0.311
Yanagihara class 2	6 (40%)	2 (8.7%)	X^2^ = 5.353, p = 0.021, phi = 0.409

*Note* Bonferroni-corrected level of significance: *p* = 0.050/3 = 0.016. Abbreviations: AN: Anorexia Nervosa; HC: healthy controls; VFI = Vocal Fatigue Index; AVQI = Acoustic Vocal Quality Index; MPT = maximum phonation time; F0 = fundamental frequency. Statistically significant differences are marked in bold and with an asterisk (*)

## Discussion

The present study represents the first research to investigate the voices of adolescents with AN, compared to a group of HC, with a non-invasive and systematic assessment and screening for the potentially confounding effect of weight measures.

Due to potential confounding factors, our search across various databases for studies comparing voice disorders to other conditions related to malnutrition or starvation yielded no relevant articles. However, dysphonia in AN can result in social withdrawal, heightened emotional distress, and further deterioration of overall health, highlighting its pivotal role in human interaction. The observed voice disorders in AN patients underscore the importance of assessing vocal changes in comprehensive management to enhance their quality of life and social well-being. Administering the VFI was crucial in understanding how vocal impairment affects daily life, as even minor voice changes can significantly impact confidence levels and communicability. Voice fatigue and weaker, breathy voices pose challenges to vocal effort, particularly in AN, where effective communication is vital for therapy and self-advocacy. Addressing these issues is essential not only for improving voice quality but also for fostering self-expression and involving individuals in the recovery process, emphasizing the significance of voice therapy as a complement to comprehensive AN treatment.

In terms of voice measures, our study added to the existing body of literature by providing valuable insights into the vocal characteristics of young people with AN compared to a control group. While neither group had moderate or severe vocal impairment on the GIRBAS scale, people with AN had a statistically higher frequency of mild impairment in areas like global dysphonia, vocal instability, and breathiness. These findings suggest that even in the absence of severe vocal pathology, there may be subtle differences in voice quality in people with AN, which expands the findings of Maciejewska and colleagues [5] regarding changes in voice parameters in this population. The authors assessed 41 girls aged 12–19 with AN, with no control group reporting voice changes mainly weak, asthenic (70.73%) and with the feature of puffing perceived in voice (41.46%). The significant differences between AN and HC in our study may add significant information to this data, but prospective research to detect potential longitudinal changes across treatment interventions is required.

One notable aspect of our study was the comparison of fundamental frequency (F0) between the two groups. Although the mean F0 values were comparable, this finding remained consistent even after controlling for %BMI. This implies that the fundamental frequency of the voice may not be significantly influenced by the diagnosis of AN itself but rather by factors related to nutritional status, contrasting the suggestion by Garcia-Santana and colleagues [12] that alterations in voice production may be linked to exposure to gastric acid during episodes of vomiting, which is more prevalent in AN. The authors assessed children and adolescents with restrictive AN and HC, documenting that patients with AN exhibited a higher mean pitch (a higher-pitched voice) than HC. Nonetheless, this research included individuals with AN with a wide age range, concerning potential puberty-related changes (which the authors carefully accounted for) (9–17; our study has a range of 12.6–16.6 years). Specific studies on puberty-related changes in individuals who develop AN would represent a clinically relevant research line.

The association between temperamental traits in individuals with AN and those with functional voice disorders may involve shared psychological characteristics such as high perfectionism, anxiety, and elevated harm avoidance. Perfectionism is a well-documented trait in eating disorders, which could exacerbate functional voice disorders by heightening concerns about voice quality to levels of significant distress and avoidance behaviors [27]. Fairburn suggested that addressing these core traits through Cognitive-Behavioral Therapy (CBT) may offer an effective approach to treating functional voice disorders by modifying perfectionist attitudes and reducing anxiety [17]. Moreover, stress management and mental health play crucial roles in therapeutic interventions for functional voice disorders [28].

Regarding acoustic voice quality, our study revealed that individuals with AN exhibited higher AVQI scores than healthy controls. Given the limited existing literature, the use of AVQI appeared to be the most suitable option for providing an overall assessment of voice quality in our preliminary study. However, given our current understanding of voice impairment in AN patients, the Acoustic Breathiness Index emerges as a promising tool for future investigations aiming to comprehensively evaluate voice parameters. However, no statistically significant difference in the frequency of over-threshold AVQI alterations was found between the two groups. Notably, a significant correlation was discovered between AVQI and %BMI, indicating that nutritional status may indeed influence voice quality. This echoes the complex relationship proposed by Ferreira and colleagues [29] and Weterle-Smoliska and colleagues [27] between FED, reflux diseases, and vocal disturbances. Notably, in our work, this correlation, as well as that concerning RSI and %BMI, resulted in significance only in the whole sample, while non-significant when run on AN patients only, removing HC. This may indicate an effect of the markedly different %BMI scores of the two groups (AN and HC), potentially limiting the clinical relevance of these findings.

In addition, our research looked into MPT, a measure of vocal endurance. Although the mean MPT values were comparable between AN and HC, it is worth noting that pathologic MPT (MPT 10 s) was only found in AN patients. This suggests that, while overall MPT may not differ significantly, people with AN may be more prone to pathologic MPT, which could be related to vocal function. This observation highlights the importance of a nuanced understanding of vocal characteristics in AN, as emphasized by Maciejewska et al. [5], who discovered differences in vocal parameters between healthy and AN individuals. The inclusion of athletes in the comparison with individuals with AN raises important considerations regarding potential confounding variables, particularly in the interpretation of MPT scores. It’s worth noting that increased cardiovascular training, common among athletes, can impact MPT scores. Future studies may benefit from including a control group of non-athletes to address this potential bias. Furthermore, the observed association between increased cardiovascular training and decreased VFI suggests the need to consider factors beyond vocal health alone when interpreting VFI differences observed in this study.

Finally, the VFI was used in our study to assess voice-related impairment. Individuals with AN had higher mean VFI scores across all three questionnaire subscales, indicating greater voice-related impairment in this population. Pathologic scores were also higher in the AN group, especially for VFI-1 and VFI-2. These findings are consistent with previous research [5]) that found voice-related changes in people with AN and suggest that these changes can have a significant impact on their daily lives.

Finally, our study adds to the growing body of literature by conducting a thorough examination of vocal characteristics in people with AN. The findings highlight the complexities of the relationship between eating disorders, reflux symptoms, and voice changes, emphasizing the need for multidisciplinary approaches to address the unique vocal challenges faced by people with AN, particularly those with low BMI and pronounced reflux symptoms. Nonetheless, more research is needed fully understand the underlying mechanisms of these voice changes and their clinical implications.

Our study found significant differences in RSI scores between people with AN and people in the HC group, with people with AN reporting significantly higher RSI scores. This finding is consistent with other studies that show a higher prevalence of GERD symptoms in people with eating disorders [30]. Furthermore, there is a significant correlation between RSI scores and %BMI. The negative correlation implies that as %BMI falls, RSI scores rise, emphasizing the importance of weight and nutritional status in reflux symptoms. This is consistent with previous research that has highlighted the role of low BMI in exacerbating GERD symptoms in people who have eating disorders [30, 31].

Upon review of our study, it became evident that while the RSI was utilized as a tool in our methodology, its direct correlation with voice characteristics might not be immediately apparent. Instead, its inclusion was strategic, aiming to capture a comprehensive profile of factors potentially influencing voice disorders in individuals with AN. This decision stemmed from emerging evidence indicating a significant association between GERD and voice alterations, particularly LPRD, which can induce dysphonia by exposing the vocal folds to acidic gastric contents, leading to inflammation. Thus, our assessment of GERD symptoms was a proactive endeavor to explore all plausible physiological factors contributing to voice changes in AN patients, beyond the disorder’s primary scope. The results highlight the intricate interplay between GERD, voice characteristics, and AN, emphasizing the necessity of a holistic approach that encompasses various physical health issues for effective clinical management. These findings contribute to a deeper understanding of the multifactorial nature of voice changes in individuals with AN, underscoring the importance of integrated treatment strategies. We acknowledge the potential influence of reduced nutritional status on the increased RSI scores observed in individuals with AN. While reflux has traditionally been associated with voice disorders in AN, this alternative perspective highlights the multifactorial nature of voice symptoms in this population. Further exploration into the nuanced relationship between nutritional status, reflux, and voice dysfunction is warranted to enhance our understanding and inform more targeted interventions for individuals with AN.

This research has some notable strengths. For starters, it takes a multidisciplinary approach to ensure standardized treatment and adherence to international clinical guidelines for people with AN and HC. By minimizing variations in treatment protocols, this approach improves the study’s internal validity. Furthermore, strict inclusion criteria for age, gender, and body weight contribute to a homogeneous study population, allowing for accurate group comparisons and reducing potential confounding variables. Furthermore, the study incorporates noninvasive evaluator-based assessments, subjective questionnaires, and software-based voice analysis. This multifaceted approach improves the tolerability for AN patients and controls, and the reliability of findings by providing a more complete picture of vocal characteristics, reducing the risk of bias associated with single assessment methods.

Despite these advantages, there are a few drawbacks to consider. The study’s small sample size limits its generalizability and statistical power, particularly for subgroup analysis within the AN population. While the cross-sectional design is useful for preliminary research, it limits the ability to assess longitudinal vocal changes in AN and provides no insight into the progression of these changes over time. Furthermore, recruiting HC from a local basketball team introduces selection bias, which may have an impact on the external validity of the study’s findings. The use of %BMI as a confounder may not fully capture the complexities of nutritional status in AN, implying that additional dietary assessments are required. The omission of endocrine blood samples, including hormones such as estrogen and cortisol, from the study was due to the focus on employing non-invasive tools for voice analysis. Given the study’s design and scope, invasive investigations such as blood tests were not included to minimize participant burden and ensure feasibility. However, future research could explore the role of endocrine dysfunction in dysphonia among individuals with AN through comprehensive endocrine assessments, offering valuable insights into potential hormonal influences on vocal function and quality. While acknowledging the limitations of relying solely on self-reported questionnaires for evaluating reflux symptoms, particularly in adolescents, the study opted for non-invasive methods to minimize participant burden. However, the absence of more objective measures like esophagogastroduodenoscopy or pH monitoring may restrict the depth of understanding regarding the relationship between reflux symptoms and dysphonia in adolescent girls with AN. Future research could consider incorporating a combination of self-reported measures and invasive techniques for a comprehensive evaluation. While our study employed statistical methods capable of identifying linear and monotone associations, it is important to acknowledge this limitation in the scope of our analyses. Non-linear relationships and complex interactions may exist within the data, which our methods were not designed to capture. Future research utilizing more sophisticated analytical techniques could provide deeper insights into these nuanced associations. Finally, the study only includes females, limiting its applicability to males with AN.

As a further limitation, although we acknowledge the importance of power analysis in determining sample size, our study, being exploratory in nature, did not incorporate a power analysis, considering the absence of prior data on voice characteristics in individuals with AN. Given the pilot nature of our study, we aimed to provide preliminary insights into voice characteristics rather than conduct a definitive analysis. Future research should aim to address these limitations for a more comprehensive understanding of vocal characteristics in individuals with AN and their potential implications for diagnosis and treatment.

## Conclusions

To summarize, this is the first study to compare the vocal profiles of adolescents with AN to a control group using non-invasive assessments and controlling for weight-related factors. We discovered subtle but significant vocal differences, implying that even mild vocal impairment exists in AN, which may be influenced more by nutritional status than by the diagnosis itself. We establish a link between nutritional status and vocal changes by correlating voice quality and %BMI. Furthermore, our study investigates vocal endurance and voice-related impairment, revealing that AN patients may be more prone to pathological vocal endurance and have more voice-related impairments, emphasizing the multifaceted nature of vocal changes in AN. We also emphasize the intricate relationship between eating disorders, reflux symptoms, and voice changes. Further research is needed to unravel the underlying mechanisms and clinical implications of these voice changes.

## Data Availability

Data will be available from the corresponding author upon reasonable request.
